# Development of Chromosome Segment Substitution Lines (CSSLs) Derived from Guangxi Wild Rice (*Oryza rufipogon* Griff.) under Rice (*Oryza sativa* L.) Background and the Identification of QTLs for Plant Architecture, Agronomic Traits and Cold Tolerance

**DOI:** 10.3390/genes11090980

**Published:** 2020-08-22

**Authors:** Ruizhi Yuan, Neng Zhao, Babar Usman, Liang Luo, Shanyue Liao, Yufen Qin, Gul Nawaz, Rongbai Li

**Affiliations:** State Key Laboratory for Conservation and Utilization of Subtropical Agro-Bioresources, College of Agriculture, Guangxi University, Nanning 530004, China; 1717303012@st.gxu.edu.cn (R.Y.); nengzhao@st.gxu.edu.cn (N.Z.); babarusman119@gmail.com (B.U.); 1717303001@st.gxu.edu.cn (L.L.); 1817303014@st.gxu.edu.cn (S.L.); qyf@st.gxu.edu.cn (Y.Q.); gulnawazmalik@yahoo.com (G.N.)

**Keywords:** rice, chromosome segment substitution lines (CSSLs), quantitative trait locus (QTL), marker-assisted selection (MAS), cold tolerance (CT)

## Abstract

Common wild rice contains valuable resources of novel alleles for rice improvement. It is well known that genetic populations provide the basis for a wide range of genetic and genomic studies. In particular, chromosome segment substitution lines (CSSLs) ais a powerful tool for fine mapping of quantitative traits, new gene discovery and marker-assisted breeding. In this study, 132 CSSLs were developed from a cultivated rice (*Oryza sativa*) cultivar (93-11) and common wild rice (*Oryza rufipogon* Griff. DP30) by selfing-crossing, backcrossing and marker-assisted selection (MAS). Based on the high-throughput sequencing of the 93-11 and DP30, 285 pairs of Insertion-deletions (InDel) markers were selected with an average distance of 1.23 Mb. The length of this DP30-CSSLs library was 536.4 cM. The coverage rate of substitution lines cumulatively overlapping the whole genome of DP30 was about 91.55%. DP30-CSSLs were used to analyze the variation for 17 traits leading to the detection of 36 quantitative trait loci (QTLs) with significant phenotypic effects. A cold-tolerant line (RZ) was selected to construct a secondary mapping F_2_ population, which revealed that *qCT2.1* is in the 1.7 Mb region of chromosome 2. These CSSLs may, therefore, provide powerful tools for genome wide large-scale gene discovery in wild rice. This research will also facilitate fine mapping and cloning of QTLs and genome-wide study of wild rice. Moreover, these CSSLs will provide a foundation for rice variety improvement.

## 1. Introduction

Wild rice (*Oryza rufipogon* Griff.) contains many novel and useful alleles that control tiller number, shattering, dormancy, pericarp color, mating type, panicle architecture and grain size and number [1]. Therefore, the potentially beneficial genes in wild rice are an important goal to improve cultivated rice (*Oryza sativa* L.) [2]. Although many quantitative trait loci (QTLs), for plant architecture, agronomic traits and cold tolerance (CT) have been identified in rice [3,4,5], however, there are few reports on those traits that were discovered in same chromosome segment substitution lines (CSSLs). The hybridization between *Oryza sativa* and wild rice and use of marker-assisted selection (MAS) of individuals retaining a part of the wild chromosome in the background revealed the transfer desirable genes to cultivated rice [6]. QTL identification through CSSLs is advantageous because it completely removes the genetic background interference and provides QTL visualization as a single Mendelian factor [7]. The development of CSSLs is laborious and time-consuming, but it is useful to scientists and plant breeders [8,9,10]. To date, several CSSLs in rice have been developed and many QTLs for traits of biologic and economic interest have been mapped [8]. The potential of the rice progenitor as a genetic resource has been explored for improving O. *sativa* with 33 chromosome segment substitution lines (CSSLs) of O. *rufipogon* (W0106) [9]. Single segment substitution lines (SSSLs) libraries also has been utilized to detect several QTLs related to plant height, heading date, seed setting rate and 1000-grain weight [10]. These achievements have undoubtedly enhanced the understanding of complex traits and encouraged plant genomic studies.

Some QTLs and genes for plant architecture traits and agronomic traits have also been detected from CSSLs [11,12]. *GL4* can control the grain length and seed shattering ability of African cultivated rice by regulating the longitudinal cell elongation of the outer glume and inner glume [11]. *RLS3* plays an important role in regulating chloroplast degradation and the normal growth of rice [12]. The additive effect, main effect and epistatic effect of QTLs were also studied based on CSSLs. In a previous study, eight SSSLs as experimental material were utilized to estimate the additive and dominant effects of six QTLs (*Hd1, Ehd1, Hd3a or RFT1, EH3, OsMADS50* and *DTH8*) and their epistatic effects among dual QTLs [13].

Cold injuries have been observed at many stages of growth and may result in the failure of germination, retarded seedling growth, stunting, discoloration, panicle tip degeneration, a prolonged duration of cultivation, sterility and irregular maturity [14]. Previous studies identified strong interactions between the cold tolerant QTLs and their environments [15,16]. Therefore, the screening of cold-tolerant germplasm and identifying the QTLs related to cold tolerance will help reduce the losses caused by low temperatures and improve rice production in marginal lands.

In this study, a broad population of DP30-CSSLs was constructed via the backcrossing, selfing and masker-assisted selection (MAS) of cultivated rice (93-11) and Guangxi wild rice (*Oryza rufipogon* Griff. DP30). By performing high-throughput whole genome sequencing, we designed 285 InDel molecular markers that were evenly distributed across the twelve chromosomes of rice. These molecular markers were utilized to select 132 substitution lines through MAS. The rate of coverage of the substitution segments to the whole genome of DP30 wild rice was 91.55%. Meanwhile, we investigated eighteen traits found in the CSSLs. thirty-six QTLs, some of which have been found in previous studies while others are new. Furthermore, we selected a cold-tolerant line (RZ34) to construct a secondary population and *qCT2.1* was located in a 1.7 Mb region on chromosome 2.

## 2. Materials and Methods

### 2.1. Plant Materials

The DP30-CSSLs were developed by using the elite rice cultivar 93-11 as the recurrent parent. Guangxi common wild rice DP30 was collected from Nanning, Guangxi Province and used as the donor parent. The original habitat of DP30 was conserved during the collection of the wild rice germplasm. Refer to previous studies to reduce the impact of environment factors on QTL, materials including receptor 93-11 and CSSLs were grown in both seasons of 2018 (fall and spring) [13].

### 2.2. Phenotyping

The phenotypic data were recorded under natural conditions in the experimental area of Guangxi University (Guangxi, China; 22°38’ N, 108°13’ E) in spring (from March to July) and fall (from July to November). A randomized complete block design (RCBD) was used with three replications for CSSLs, CSSL combinations and their parents. The parent plants and 20 CSSL plants were selected from each replication for data collection. The data for 17 traits were recorded, including leaf sheath color (LSC), leaf margin color (LMC), tiller angle (TA), heading date (HD), plant height (PH), grain shattering (SH), apiculus color (AC), stigma color (SC), glume color (GC), number of grains per panicle (NGPP), 1000-grain weight (GWT), grain length (GL), grain width (GW), grain length to width ratio (GLWR), awn length (AL), seed coat color (SCC) and cold tolerance (CT). The data for TA was recorded on a scale from 1 to 9 representing the angles of 0–10°, 11–20°, 21–30°, 31–40°, 41–50°, 51–60°, 61–70°, 71–80° and 81–90°, respectively [17,18,19,20]. The HD was recorded when the first panicle to emerge reached about 2-cm-long and the number of days from sowing to heading were scored for each plant. PH was measured for each plant at the mature stage from the base of the stem to the tip of the higher panicle. NGPP, GL and GW were recorded according to the previously established methods [19,20]. The GLWR and 100-GWT of the filled grains were investigated after the rice was harvested at the mature and naturally dried stage [10].

### 2.3. Evaluation of Cold Tolerance at Seedling Stage

To preliminarily evaluate the cold tolerance variation of CSSLs, the experiment was conducted in controlled conditions at seedling stage. Thirty seedlings of each CSSL were planted in soil. Plants were grown in a controlled environment at a day temperature of 25 °C and night temperature of 19 °C till three leaf stage. At three leaf stage, seedlings were exposed to cold stress according to a previously established method [21]. The depth of the water was about 5 cm measured from the surface of the soil in the trey. The cold stress treatment lasted 5 days and the conditions alternated between 10 °C for 10 h during the day and 8 °C for 14 h at night. After the cold treatment, the seedlings were subjected to natural standard growth conditions at 26 °C and the survival rate was investigated. After 5 days of treatment, cold tolerance was evaluated on the basis survival rate and injury level. The experiment was repeated three times under the same cold stress treatment. The average data of three replications were used. The data for CT were recorded on a scale from 0 to 9, representing the survival rates of 0–9%, 10–19%, 20–29%, 30–39%, 40–49%, 50–59%, 60–69%, 70–79%, 80–89% and 90–100%, respectively [21]. The secondary mapping population used to fine-map the major QTL for cold tolerance was developed by backcrossing a CSSL(RZ34) with the recipient parent (93-11). Three hundred and eleven F_2_ plants of that cross were genotyped using five InDel markers and fifty-seven cold tolerant and twenty-one cold sensitive plants selected for phenotypic evaluation under cold stress treatment.

### 2.4. Construction of CSSLs and Genome Sequencing and Development of InDel Markers

CSSLs of common wild rice were constructed by hybridization, backcrossing and marker-assisted selection (MAS) according to the previously described method [22,23]. The genomic DNA of DP30 and 93-11 was prepared and whole-genome re-sequencing (WGRS) was performed on an Illumina HiSeq2500™ by Novo Generation Company, Beijing, China. The standard Illumina protocol was followed for sample preparation and sequencing. The quality trimming (phred quality score, <Q_20_) was carried out by using FastQC [24] and the Cutadapt software was used for adapter trimming with the parameters of −O 5 and −m 32 [25]. The Burrows–Wheeler Aligner (BWA) software was used to map clean reads to the 93-11-reference genome [26,27]. InDel polymorphisms were detected by the GATK tool software with the defined length of insertions and deletions between 1 bp and 10 bp [28]. The larger sized (≥2 bp) InDel regions and high sequencing depths (DP, ≥5-fold) were extracted to design the InDel markers. The primer pairs were designed based on parental sequence differences by the DNAMAN v6.0 software and screened using the NCBI (https://www.ncbi.nlm.nih.gov/) database.

### 2.5. DNA Isolation and PCR Amplification

Genomic DNA was extracted from fresh leaf tissues using the CTAB method described by the previously established protocol [29]. The PCR amplification, separation of PCR products and confirmation and genotyping of the electrophoretic bands of the PCR products were performed according to previously established methods [30,31,32].

### 2.6. QTL Mapping and Data Analysis

The lengths of the substituted segments in CSSLs were assayed according to the previously established method [10]. A chromosomal segment flanked by two donor markers was considered to be 100% donor type, and the length of the segment was considered to be the minimum length of a substituted segment (L-min). A chromosome segment flanked by two recipient markers was considered as 0% donor type, and the length of the segment was considered to be the maximum length of a substituted segment (L-max). A chromosome segment flanked by one marker of donor type and one marker of recipient type was recognized as 50% donor type. The length of each substituted segment in the CSSLs was calculated as the estimated length (L), which is the average of L-min and L-max.

Genotypic graphics and chromosome genetic maps of the CSSLs were generated using the Graphic Geno-Types 2 software (GGT2.0) [10]. A putative QTL was declared at the significance level of *p* ≤ 0.001 in a CSSL. If several CSSLs with overlapping substituted segments shared the same QTL, a substitution mapping approach was employed to localize the QTL to a smaller genomic interval [33]. QTL nomenclature was performed according to McCouch and CGSNL: each QTL name is italicized and starts with a lowercase letter “q” to indicate that it is a QTL, followed by a two to five letter standardized “trait name”, a number designating the rice chromosome on which it occurs (1–12), a period (“.”) and a unique identifier to differentiate individual QTLs for the same trait that resides on the same chromosome [34]. The linkage map of QTLs was constructed using MapChart 2.2 [35]. The statistical analysis of the phenotypic and genotypic data of the DP30-CSSL populations was performed by QTL IciMapping 4.1.0 software [36]. Base on the permutation test to set LOD value ≥2.5 as the thresholds for QTL analysis. The chromosomal genetic map was constructed using MapChart 2.3 software.

The genetic effect of different QTLs according to the previously established following formulas [13]:

(1) Additive effect (a) = phenotypic value of CSSL (CSSL) − phenotypic value of 93-11(93-11);
(2) Dominant effect (d) = 93-11 × CSSL − 93-11.

## 3. Results

### 3.1. Phenotypic Variation of Plant Architecture, Agronomic Traits and Cold Tolerance in DP30-CSSLs

We evaluated the variation in plant architecture, agronomic and cold tolerance traits (DP30, DP30-CSSLs and 93-11) and calculated the phenotypic values of these traits during two seasons (fall and spring). The DP30-CSSLs showed significant variation in all of these traits. Furthermore, this variation in HD, GWT, GL, GW, GLWR and AL were normally distributed, while TA, PH, NGPP, SH and CT followed biased distribution (Figure S1). First, we speculated that the biased distribution was due to continuous backcrossing, that the CSSLs without related trait substitution segments were similar to 93-11. Second, we counted 132 lines, so an insufficient population may have led to biased distribution. Third, these distributions were frequently skewed because of interactions between the alleles, nonallelic genes or other environmental factors.

### 3.2. Genome Re-Sequencing and Selection of InDel Markers

**Figure 1:**
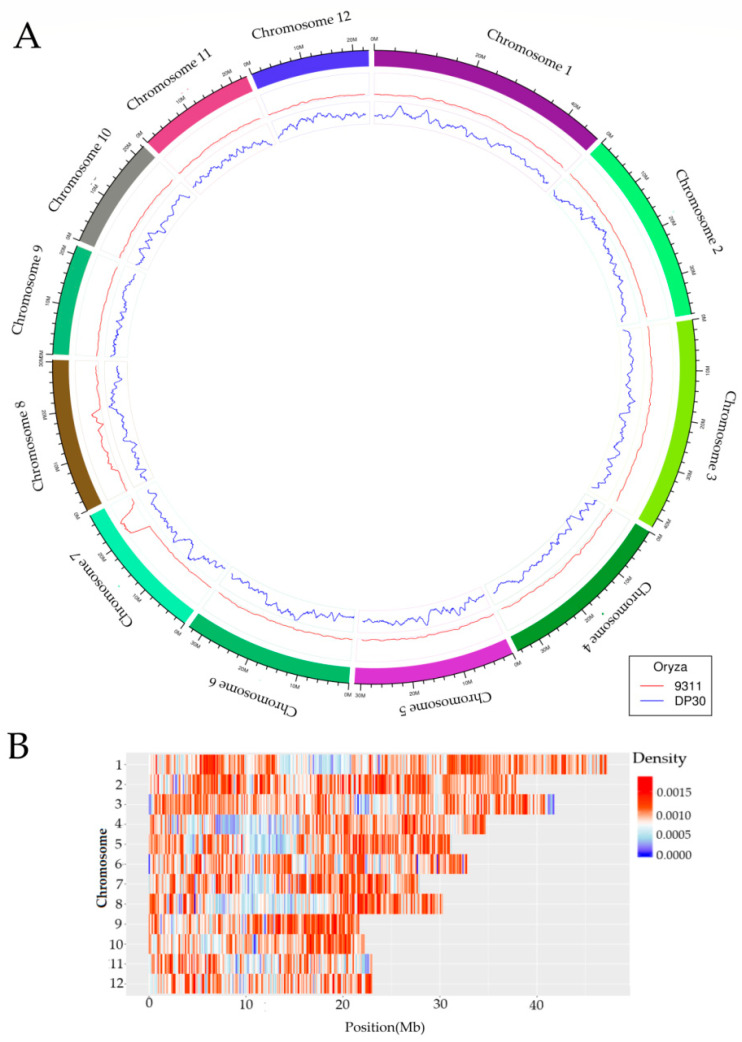
Whole-genome sequencing of DP30 and genome-wide sequence difference. (**A**) Circos plot for SNPs and InDels detected in 93-11 and DP30. The outermost circle depicts the ideogram of the 12 chromosomes in the rice genome. The innermost lines represent the frequency SNPs and InDel markers identified between DP30 (blue) and 93-11 (red); (**B**) genome-wide distribution of sequence differences between 93-11 and DP30. Red and blue colors represent the degree of variation between DP30 and 93-11. Depth of the color indicates the level of variation frequency.

The DP30 genome was sequenced by Illumina high-throughput sequencing technology, and the resulting sequence was assembled using IRGSP-1.0, with the 93-11 genome as a reference (Figure 1A). The DP30 genome is displayed according to the input reads (Table S1). The sequencing results reveal that 1,894,103 bp across all 12 chromosomes were different in DP30 and 93-11, representing 6.1% of the genome (Figure 1B). InDel molecular markers were designed such that the base mutation exceeded 20 bp. Based on the lengths of the PCR products (150–200 bp) and the results of gel electrophoresis, we selected 285 InDel markers with an average distance of 1.2 Mb (Table 1). The sequences of the 285 InDel markers (Table S2).

### 3.3. Development and Substitution Segment Analysis of DP30-CSSLs

#### 3.3.1. Development of the DP30-CSSLs

**Figure 2:**
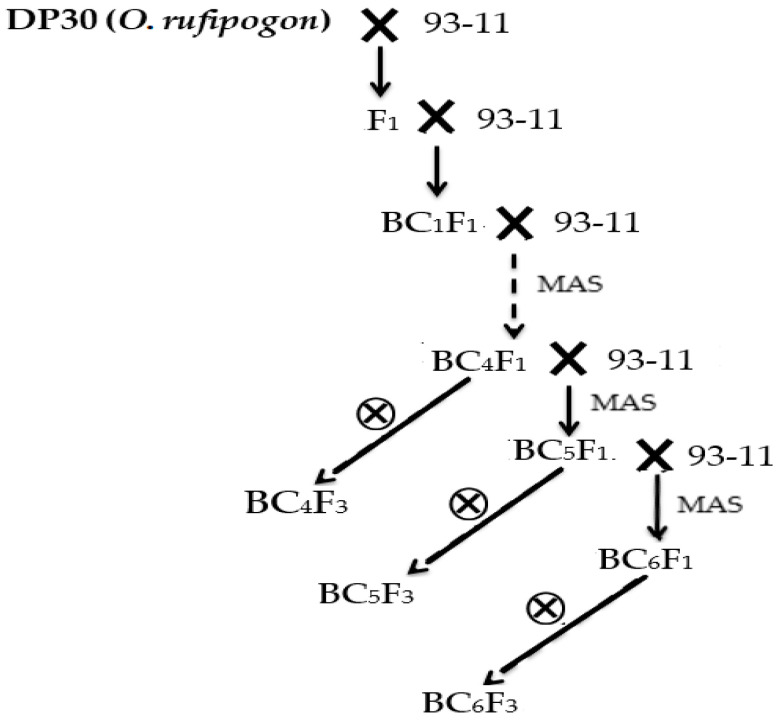
Schematic of the process used to develop DP30-CSSLs in this study. MAS—molecular marker-assisted selection; ×—hybrid; ⊗—self-crossing.

The procedure we developed the DP30-CSSLs is shown in Figure 2. The F_1_ plants were obtained by crossing DP30 and 93-11. Molecular MAS began using the BC_4_F_1_, resulting in 176 BC_5_ F_3_ lines, 32 BC_6_ F_3_ lines and 22 BC_7_ F_3_ lines containing the target segments. Finally, we selected 132 CSSLs from these 230 lines (candidate plants) for genetic and phenotypic analysis (Figure 2, Table S3).

#### 3.3.2. Substitution Segments of DP30-CSSLs

**Figure 3:**
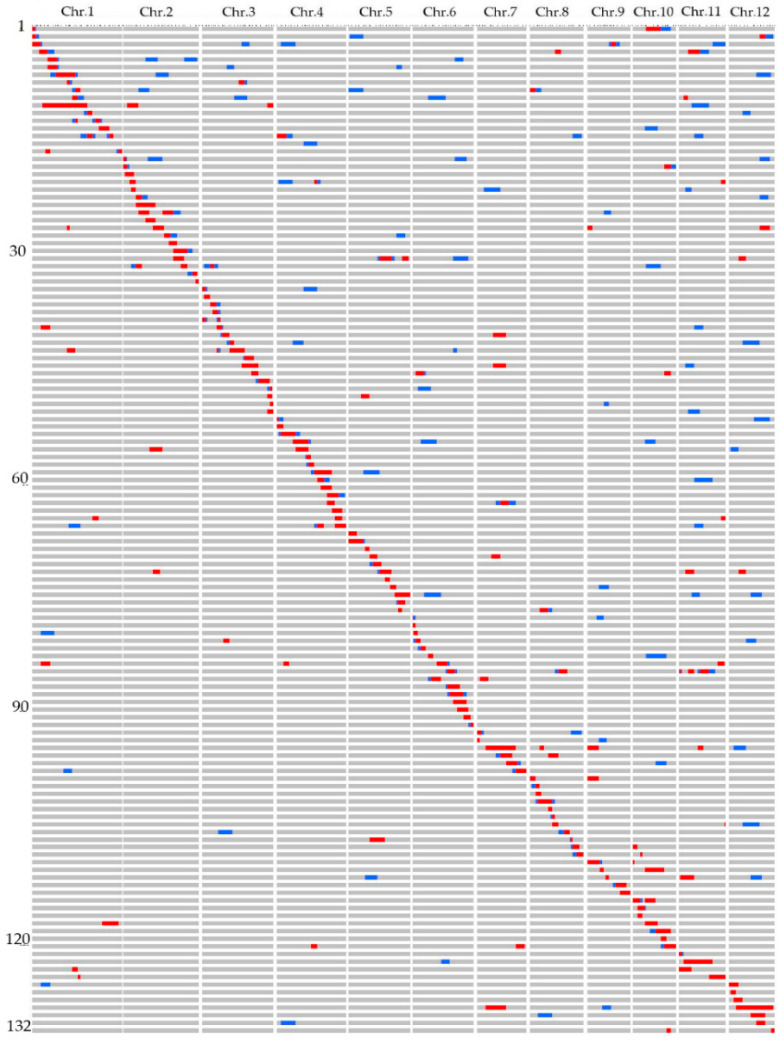
Graphic genotypes of the 132 DP30-CSSLs developed in this study. Red bars indicate homozygous substituted segments derived from DP30. Blue bars indicate heterozygous substituted segments derived from DP30. Gray bars indicate the genetic background of the recipient parent 93-11, “Chr.”—chromosome.

In total, we identified 80 BC_4_ F_2_ plants from 171 CSSLs and named as DP30-CSSLs. Then, 24 BC_4_ F_2_ plants were selected and 44 BC_5_ F_2_ plants were obtained after continuous backcrossing. Finally, 18 BC_6_ F_2_ were obtained with a relatively complex genetic background to continue backcrossing. The 132 CSSLs formed a DP30-CSSLs population, and the target replacement segments of these CSSLs accumulated a total length of 536.4 Mb, covering 91.42% of the DP30 genome. The CSSLs were arranged according to the position of their target substitution segments. The 99 substitution lines contained only one substitution segment from DP30. The total length of these CSSLs substitution segments was 359.66 Mb and the coverage rate was 61.36% (Figure 3).

Chromosomes with more than 90% coverage of the DP30 genome by chromosome substitution segments include chromosomes 1, 2, 3, 5, 7, 11 and 12. Among them, chromosome 5 had the highest coverage rate (97%), while the lowest coverage rate belonged to chromosome 8 (Table 2).

The average length of DP30-CSSLs population substitution target segment was 4.06 Mb, which ranges from 0.5–22 Mb. The length of the substitution segment of 25 CSSLs was less than 2 Mb; The length range of substitution segments of 55 substitution lines was 2–4 Mb and 30 CSSLs were 4–6 Mb and the length of the substitution segment of 6 CSSLs was larger than 8 Mb.

### 3.4. Wild rice QTLs in the DP30-CSSLs

We detected the genotypes of all CSSLs in DP30-CSSLs (Table S3). In total, 36 QTLs were identified in the CSSLs.

#### 3.4.1. Tiller Angle (TA) and Heading Date (HD)

Four QTLs (*qTA1.1, qTA7.1, qTA9.1* and *qTA2.1*) related to TA were detected in the CSSLs. Seven CSSLs, (RZ18, RZ20, RZ38, RZ104, RZ129, RZ130 and RZ168) had different TAs those of 93-11 (Figure S2A,B). *qTA1.1* was located in the overlapping segment of RZ18 and RZ20, which belong to C1-26–C1-27 in chromosome 1. *qTA7.1* was detected in an overlapping segment of RZ104 and RZ168 that located in C7-2 on chromosome 7, the *PROG1* was also in the same position of chromosome [17]. *qTA9.1* and *TAC1* were detected in overlapping segments of RZ129 and RZ130, which was in the C9-14–C9-15 region of chromosome 9 [20] (Table 3). The TAs of the RZ38 plants were between 0° to 10°, while 93-11 plants had TAs of 30° to 15°, in both seasons (Figure S1A,B). The QTL *qTA2.1* in the segment of RZ38 was located near the molecular marker C2-25 on chromosome 2 (Table 3; Figure S6).

Three CSSLs (RZ142, RZ8 and RZ13) showed significant differences in HD when compared with 93-11 (Figure S2C). These three lines had an overlapping segment near C11-4 on chromosome 11, so we identified a new QTL (*qHD11.1*) in this region that has negative additive effects on HD (Table 3; Figure S6).

#### 3.4.2. Plant Height (PH)

We found three QTLs (*qPH1.1, qPH6.1* and *qPH7.1*) related to PH in the CSSLs, which were distributed on chromosome 1, 6 and 7, respectively. Five CSSLs (RZ14, RZ16, RZ17, RZ92, RZ105, RZ107 and RZ164) had PH values of 170 ± 9 cm, while 93-11 plants were about 110 ± 5 cm (Figure S2D). *qPH1.1* was identified in the overlapping segment of RZ14, RZ16, RZ17 and the *Sd1* was in the same position [37]. The RZ92 and RZ164 segments overlapped near the molecular marker C6-8 on chromosome 6 and the *qPH6.1* was identified in this region. The *qPH7.1* was identified in the overlapping segments of RZ105 and RZ107 near the molecular marker C7-5 on chromosome 7 (Table 3; Figure S6).

#### 3.4.3. Number of Grains per Panicle (NGPP) and 100 Grain Weight (GWT)

**Figure 4:**
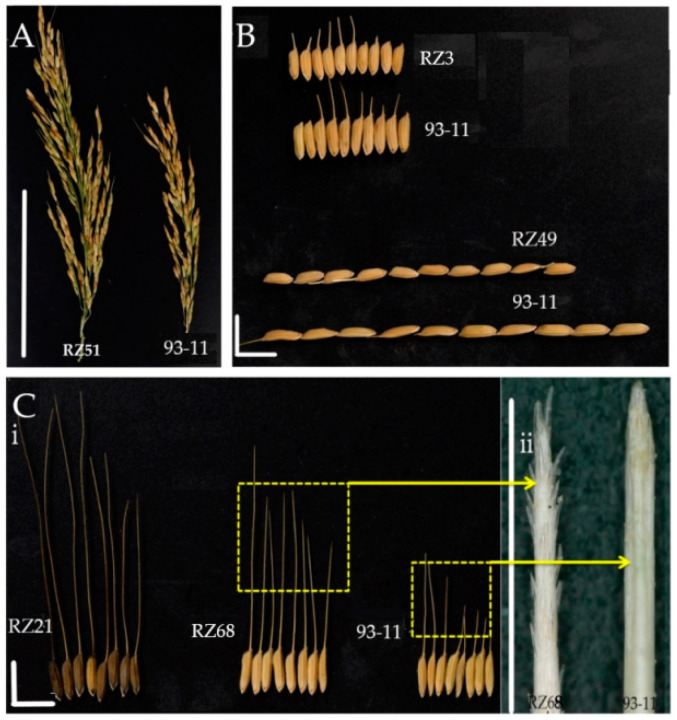
Phenotypic variation in the appearance of grains and awns in CSSLs and 93-11. (**A**) Grain number per ear for RZ50 and 93-11 (bar = 10 cm); (**B**) length of grains of RZ3 and 93-11 (bar = 1 cm); (**C**) Phenotypes of the awns of RZ21, RZ68 and 93-11: (**A**) awn length (bar = 1 cm) and (**C**) awn tip surfaces of RZ68 and 93-11 (bar = 1 mm).

We identified three QTLs (*qNGPP3.1, qNGPP4.1* and *qNGPP12.1*) related to NGPP that were distributed on chromosome 3, 4 and 12. Seven CSSLs (RZ51, RZ50, RZ52, RZ64, RZ20, RZ144 and RZ145) had more than 210 NGPP while 93-11 had less than 180 NGPP in both seasons (Figure 4A). We identified QTL *qNGPP3.1* in C3-19 and *qNGPP4.1* in C4-12. An overlapping segment in C12-2 contained a QTL named *qNGPP12.1* (Table 3; Figure S6).

#### 3.4.4. Grain Length (GL), Grain Width (GW) and Grain Length to Width Ratio (GLWR)

Two QTLs (*qGL3.1* and *qGL9.1*) related to GL were detected in the CSSLs on chromosome 3 and 9. Three CSSLs (RZ3, RZ49 and RZ50) had GLs of 8.40 ± 0.06 mm, while 93-11 had a GL of 10.03 ± 0.13 mm (Figure 4B). The RZ49 and RZ50 segments overlapped near the molecular marker C3-19 on chromosome 3 and this new QTL was named *qGL3.1*. The QTLs *qGL9.1* and *SG1* were detected in the segment of RZ3 near the molecular marker C9-12 on chromosome 9 [39] (Table 3; Figure S6).

Two QTLs (*qGW9.1* and *qGW10.1*) related to GW were detected on chromosome 9 and 10. Three CSSLs, (RZ133, RZ3 and RZ134) had GWs of less than 2.85 mm, while 93-11 had a GW of more than 2.98 mm (Figure 4B). The RZ3 segment was located near the molecular marker C9-8 on chromosome 9 and named *qGW9.1*. We identified *qGW10.1* in the overlapping segment of RZ133 and RZ134, near the molecular marker C10-3 on chromosome 10 (Table 3; Figure S6).

Two QTLs (*qGLWR3.1* and *qGLWR8.1*) related to GLWR were detected on chromosome 3 and 8, respectively. Three CSSLs, (RZ56, RZ111 and RZ167) had GLWRs of 3.5 while 93-11 had a GLWR of 3.3. The overlapping segments of RZ56 and RZ167 were found near the molecular marker C3-26 on chromosome 3 and named *qGLWR3.1*. We detected *qGLWR8.1* in the segments of RZ112, near the molecular marker C8-2 on chromosome 8 (Table 3; Figure S6).

#### 3.4.5. Awn Length (AL)

Five QTLs (*qAL1.1, qAL4.1, qAL4.2, qAL8.1* and *qAL11.1*) related to awn length (AL) were detected on chromosome 1, 4, 8 and 11. Twelve CSSLs (RZ21, RZ6, RZ4, RZ62, RZ63, RZ70, RZ68, RZ119, RZ4, RZ141, RZ88 and RZ142) had ALs of 30 ± 4 mm, while the AL of 93-11 was 14 ± 3 mm (Figure 4C). The overlapping segments of RZ21, RZ6 and RZ4 were found near the molecular marker C1-7 on chromosome 1 and named *qAL1.1*. The QTLs *qAL4.1* and *An-1*/allele were detected in RZ62 and RZ63 near the molecular marker C4-8–C4-10 on chromosome 4. [40] Two other QTLs, *qAL4.2* and *An-2*/allele were detected in the overlapping segments of RZ70 and RZ68 near the molecular marker C4-19 on chromosome 4 [41]. The grains of common wild rice have long and spiny awns. In contrast, cultivated rice species have no awns or short awns with smooth surfaces (Figure S4C). The overlapping segments of RZ135 and RZ4 were located near the molecular marker C8-11 on chromosome 8 and named *qAL8.1*. We found *qAL11.1* in the overlapping segments of RZ142, RZ88 and RZ141 near the molecular marker C11-2 on chromosome 11 (Table 3; Figure S6).

#### 3.4.6. Grain Shattering (SH) and Cold Tolerance (CT)

**Figure 5:**
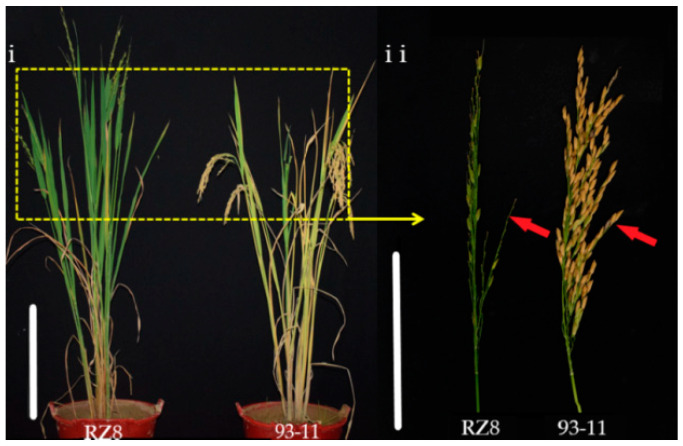
Phenotypic variation of grain shattering in RZ8 and 93-11; Whole plant phenotype showing grain shattering (bar = 50 cm), grain shattering (bar = 10 cm, red arrows).

The grains of 93-11 did not shatter in either season, but the grains of five CSSLs (RZ74, R75, RZ76, RZ8 and RZ88) did shatter (Figure 5). We found *qSH4.1* in the overlapping segments of RZ74, R75 and RZ76 near the molecular marker C4-22 on chromosome 4, the *SH4* was also in the same chromosomal position [42]. *qSH11.1* in the overlapping segments of RZ8 and RZ88 (Table 3; Figure S6).

Seven QTLs (*qCT1.1*, *qCT2.1*, *qCT3.1*, *qCT5.1*, *qCT6.1*, *qCT10.1* and *qCT12.1*) related to cold tolerance (CT) were detected on chromosome 1, 2, 3, 5, 6, 10 and 12. Seventeen CSSLs (RZ10, RZ11, RZ12, RZ34, RZ40, RZ41, RZ85, RZ86, RZ87, RZ99, RZ100, RZ101, RZ136, RZ137, RZ158, RZ162 and RZ163) were more tolerant to cold stress compared with 93-11 (Figure S3). We identified *qCT1.1* in the overlapping segments of RZ10, RZ11 and RZ12 near the marker C1-16 on chromosome 1, which also contained the gene *OsRAN1* [43]. *qCT2.1* was found in the overlapping segments of RZ34, RZ40, RZ137, RZ158 and RZ170 near the marker C2-19. *qCT3.1* was detected in the overlapping segments of RZ40 and RZ41 near marker C3-2. *Qc T5*.1 was identified in the overlapping segments of RZ85, RZ86, RZ87 and RZ170 near the marker C5-20 on chromosome 5, the *OsiSAP8* was also in the same chromosomal position [44]. The QTLs *qCT6.1* was found in the overlapping segments of RZ99, RZ100, RZ101 and RZ171 near the marker C6-20 on chromosome 6, the *OsPYL9* was also in the same chromosomal position [45]. chromosome 10 contained the QTL *qCT10.1*, which was identified in the overlapping segments of RZ136, RZ137 and RZ171 near the marker C10-3. The overlapping segments of RZ158, RZ162 and RZ163 were located near the marker C12-11 on chromosome 12 and named *qCT12.1* (Table 3).

#### 3.4.7. Identification of Loci Related to Quality Traits

In five CSSLs (RZ5, RZ112, RZ19, RZ75 and RZ106), significant variation with 93-11 in LSC, LMC, AC, SC, GC and SCC (Figures S4 and S5). Segments of these CSSLs are located in chromosomes 1, 4, 7 and 11. Six related trait genes (*LSC11.1*, *LMC11.1*, *AC1.1*, *SC1.1*, *GC4.1* and *SCC7.1*) were identified; *AC1.1* and *SC1.1* may be the same gene. *AC1.1* and *SC1.1* may be the allele gene of *A. GC4.1*, SCC7.1 and *Bh4, Rc* were in the same chromosome position [46,47,48].

### 3.5. Construction of a Secondary Population and Mapping of qCT2.1

**Figure 6:**
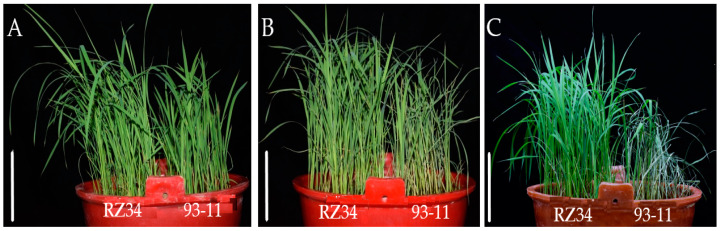
Cold stress treatment to RZ34 and 93-11 at seedling stage. (**A**) Before cold stress (bar = 10 cm); (**B**) after cold stress (bar = 10 cm) and (**C**) after three days of recovery (bar = 10 cm).

Our results showed that there was no significant difference in the appearance of RZ34 and 93-11 seedlings before cold treatment (Figure 6A). RZ34 had a lesser degree of wilting after cold treatment compared with 93-11 (Figure 6B). Furthermore, 93-11 died three days after the cold treatment, while RZ34 survived and continued to grow (Figure 6C). The results show that RZ34 seedlings were more tolerant to cold stress than 93-11. All the F_1_ individuals in the RZ34 line exhibited a cold tolerant phenotype. We selected some of the F_1_ plants and the related molecular markers showed that these plants were heterozygous for the tested genotypes. We obtained a total of 311 plants in the F_2_ population, among them 223 plants were cold tolerant, and 88 plants were susceptible to cold stress. This represented a 3:1 segregation ratio for cold tolerance which consistent with Mendelian’s rules (χ^2^ = 1.630 < χ^2^_0.05,1_ = 3.84), demonstrating that this cold tolerance trait was controlled by a single dominant QTL.

**Figure 7:**
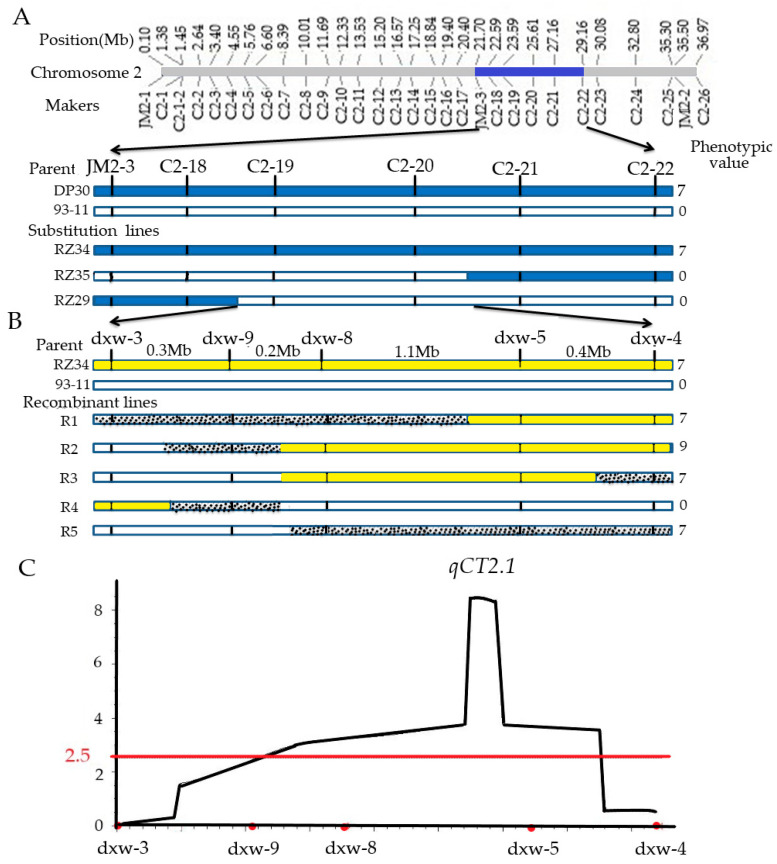
Mapping of grain-shattering QTL *qCT2.1*, (**A**) QTL analysis by overlapping segments of recombinants RZ34, RZ35 and RZ29, blue intervals indicate DP30 genotype; blank intervals indicate: 93-11 genotype; shadow intervals indicate heterozygous genotype; (**B**) linkage analysis of *qCT2.1* yellow intervals indicate RZ34 genotype, blank intervals indicate 93-11 genotype, shadow intervals indicate heterozygous genotype; (**C**) LOD value of *qCT2.1.* dxw-3, dxw-8, dxw-9, dxw-5 and dxw-4 are InDel markers.

We identified the QTL *qCT2.1* in the segment of RZ34 (Figure 7A) between markers JM2-3 (21.7 Mb) and C2-22 (29.18 Mb). Based on the tracking and overlap of three CSSLs (RZ37, RZ38 and RZ39) segments, the range of *qCT2.1* was reduced to 4.47 Mb (Figure 7A). Five InDel markers (dxw-3, dxw-9, dxw-8, dxw-5 and dxw-4) were developed between C2-18 and C2-20 (Table S4). In order to reduce phenotypic identification error and improve the accuracy of QTL mapping, we selected individual plants with extreme phenotypic values to detect genotypes. We identified the phenotypes of 311 plants in the F_2_ population. Among them, the genotypes of 56 cold-tolerant plants (scale 9–7) and 22 cold-sensitive plants (scale 0) were detected for linkage analysis. Five recombinant plants (R1, R2, R3, R4 and R5) were selected from the constructed F_2_ population (Table S5). These plants confirmed that *qCT2.1* is located in a 1.7 Mb region between molecular markers dxw-4 and dxw-9 (Figure 7B) and that the LOD value of *qCT2.1* was 8 (Figure 7C).

## 4. Discussion

### 4.1. Constructing CSSLs in Guangxi Wild Rice (*O. rufipogon* Griff.) DP30

Since QTL detection is based on the natural allelic differences between parental cultivars, it is important to select parental cultivars that show large phenotypic variation in the target traits [49]. There is rich polymorphism between Guangxi wild rice DP30 (*O. rufipogon* Griff.) and cultivated rice 93-11 (*O. sativa* L.) due to their distant genetic bases [10]. In this study, we performed MAS using Guangxi wild rice DP30 (*O. rufipogon* Griff) as a donor parent to develop 132 DP30-CSSLs. The coverage rate of the DP30 wild rice was 91.55%. The average length of each replacement segment was 0.5–22 Mb. Compared with the previously reported CSSLs in wild rice, the DP30-CSSLs had a higher substitution segment coverage rate and more polymorphic primers for MAS [50,51]. Clearly, the genomic constitution quality of the CSSLs population is important. The chromosome position and genetic effect of QTLs locating on dp30-CSSLs will be more accurately assessed.

### 4.2. Identification of Quantitative Trait Loci and Measurement of Various Traits in Fall and Spring

Previous reports have shown that the CSSLs of wild rice are effective in the mining and transferring of wild alleles into cultivated rice [10]. Furthermore, several studies have reported the development of CSSLs and the identification of several agronomic and plant architecture traits [17,35,36,37,38,39,40,41]. Ma et al. (2019) detected eighteen QTLs were two known grain length- and width-related genes and four novel QTLs. In addition, two QTLs were verified, and two novel QTLs were identified, for panicle neck length, a domestication-related trait [49]. Tan et al. (2004) identified quantitative trait loci (QTLs) associated with plant height and the days to heading in the BC_3_ F_2_ population. Putative QTLs derived from O. *rufipogon* were detected for plant height on chromosome 1 and identified 6 QTLs for days to heading on chromosomes 1, 3, 7, 8 and 11 [50].

In the present work, we compared the phenotypes of DP30-CSSLs to the phenotypes of known genes and used related molecular markers to confirm whether any of these genes/allele genes were present in the segments. We identified five QTLs related to TA, *qTA7.1* and *PROG1* were in the same position on the chromosome; *qTA9.1* may be the allelic form of *TAC1* (Table 3). *PROG1* (LOC_Os07g05900) controls the creeping growth habits of common wild rice [17]. *TAC1* (LOC_Os09g35980) is a recently discovered gene that controls the TA of rice corresponding to a major QTL [20]. We found that these five QTLs had positive additive effects on TA (Table 3). A QTL named *qHD11.1* had negative additive effects on HD. Three of the QTLs identified in this study (Table 3) had positive additive effects for phenotypic variations in PH in both fall and spring. *qPH1.1* and *Sd1* were in the same position on the chromosome (Table 3). The *Sd1* (LOC_Os01g66100) gene, which controls gibberellin biosynthesis, was among these QTLs [36]. Three QTLs showed negative additive effects on NGPP and *qGWT1.1* may be the allele of *OsAGPL2* (Table 3). *OsAGPL2* (LOC_Os01g44220) is a member of the *OsPYL* gene family that regulates the filling rate of grains, leading to lower final grain weight and yields [37]. Except for the QTLs locus near the *OsAGPL2* gene, we also identified eight other QTLs. All of them showed negative additive effects on 100GWT in both seasons (Table 3). The *SG1* (LOC_Os09g28520) gene has shorter grains than the wild type and a dwarf phenotype [39]. Similar to the *SG1 gene,* one new QTL we identified, which showed negative additive effects on GW (Table 3). We identified five AL-related QTL, *qAL4.1* and *qAL4.2* as alleles of *An-1* and *An-2*, respectively (Table 3). *An-1* (LOC_Os04g28280) regulates the formation of awn primordia, promoting awn elongation and increasing the length of grains [40]. *An-2* (LOC_Os04g43840) also increases the length of awns and makes them spinier (Figure S4) [41]. The QTL *qSH4.2*, which is related to grain shattering, the *SH4* (LOC_Os04g57530) was in the same chromosome position [42] (Table 3). Cold tolerance in seedlings is one of the important traits for the stable production of rice [43,44,45]. Here, we identified seven QTLs related to cold tolerance including these loci in a similar region with these three previously cloned genes. *qCT1.1*, *qCT5.1* and *qCT6.1* may be the allelic form of *OsRAN1*, *OsiSAP8* and *OsPYL9*, respectively (Table 3). *OsRAN1* (LOC_Os010611100) participates in cell division and the cell cycle and promotes the formation of intact nuclear membranes, thus improving the cold tolerance of rice [43]. *OsiSAP8* (LOC_Os06g41010) is a zinc finger protein gene that enhances salt, drought and cold stress tolerance in rice [44]. *OsPYL9* (LOC_Os06g33690) is a member of the *OsPYL* gene family and is a possible abscisic acid (ABA) receptor [45]. In addition, there are six quality trait loci, which contain three alleles [46,47,48]. The alleles of eleven cloned genes showed the reliability of DP30-CSSLs. No cloned genes were found in other QTL, which may contain new genes.

### 4.3. Construction of Secondary Population and Mapping of qCT2.1

Map-based cloning and mapping of cold tolerance genes in rice have always been a classical method for cold tolerance research in rice.

Previous studies used different populations to obtain some cold tolerance genes of rice [51,52]. According to the published data, more than 250 QTL of low-temperature tolerance has been found on 12 chromosomes of rice. In DP30-CSSLs, *qSCT-3-1* was identified in the RM15031-RM3400 region of the long arm of chromosome three near to the centromere, and the genetic distance between the linkage markers was found to be 1.8 cM [53]. In this study, F_2_ populations were constructed by Guangxi common wild rice seedling cold-tolerant segment replacement line RZ34 and cold-sensitive recurrent parent 93-11. Through map-based cloning, it was found that the main cold tolerance QTL *qCT2.1* of rice at the seedling stage was located on chromosome 2 and located in the range of 1.7 Mb between molecular marker dxw-4 and dxw-9. To date, there is no cloned cold tolerance gene at the seedling stage in this interval. *qCT2.1* could enhance cold tolerance at the seedling stage, which has a strong dominant effect, so it is expected to be used in rice breeding.

Rice breeding entered to a new era with the utilization of MAS and whole-genome sequencing to link genotypes with phenotypes. The introduction of wild rice CSSLs promoted gene QTLs mapping and genomic research. This study also suggests using Guangxi common wild rice accessions will provide a broad platform for genomic research and may lead to the discovery of new QTLs that will benefit rice breeding.

## 5. Conclusions

This study aims to use wild rice to develop CSSLs for cultivated rice and use these CSSLs for comparative mapping of traits related to plant architecture and yield. We focused on germplasm innovation in rice through the identification and transfer of beneficial genes/QTLs from the wild species. We introduced the DP30-CSSL library platform to facilitate pre-designed breeding of cultivated rice to utilize favorable alleles dispersed in Guangxi wild rice resources. The QTLs presented here are expected to provide further clues to identifying underlying mechanisms involved in plant architecture and improved grain. Our ongoing experiments are aimed at confirming the genomic regions and narrowing down of number of genes reported within the QTLs in the present study through comprehensive studies involving high-resolution linkage mapping via high-throughput genotyping by sequencing of advanced generation progenies.

## Figures and Tables

**Table 1:** Number of single-nucleotide polymorphism (SNPs) detected in wild rice (DP30).

Chromosome	SNP No. (bp)	Chr. Length (bp)	SNP Percentage	Length (Mb)	Marker No.
1	232,372	47,283,185	0.49%	1.32	38
2	210,389	38,103,930	0.55%	1.15	30
3	194,967	41,884,883	0.47%	1.33	32
4	155,761	34,718,618	0.45%	1.21	25
5	164,450	31,240,961	0.53%	1.34	24
6	167,985	32,913,967	0.51%	1.22	25
7	153,895	27,957,088	0.55%	1.1	19
8	154,296	30,396,518	0.51%	1.2	21
9	119,958	21,757,032	0.55%	1.26	18
10	113,121	22,204,031	0.51%	1.21	17
11	107,638	23,035,369	0.47%	1.24	18
12	119,271	23,049,917	0.52%	1.18	18
Average	157,842	31,212,125	0.51%	1.23	24
Total	1,894,103	374,545,499	6.10%		285

SNP-No—number of SNPs detected in DP30; Chr.-length (bp)—chromosome length; Length (Mb)—average length between adjacent markers in each chromosome; SNP Percentage—percentage of SNP; Marker No.—the number of molecular markers on each chromosome; Mb—million bp.

**Table 2:** Substitution segment analysis in DP30-CSSL.

Chromosome	No. of CSSLs	Total Length of Target Segment (Mb)	Length of Chromosome (Mb)	Coverage Length (Mb)	Coverage Rate (%)
1	17	72	47	44.5	94.7
2	17	61	38	36.5	96.1
3	17	52.5	41	38.1	93.3
4	15	66.6	35	31.9	91.2
5	11	41.9	32	29.4	92.3
6	15	56.8	33	28.7	87.9
7	6	31.9	28	26.6	95.5
8	11	29.6	30	24.9	83.3
9	5	21.7	22	19.1	87.5
10	7	31.6	22	19.4	88.6
11	4	26.2	23	21.4	93.1
12	7	24.9	23	21.7	93.6

CSSLs No.—number of substitution lines on chromosomes; Chr.-Length (Mb)—chromosome length; Coverage length (Mb)—covering length of the substitution segment to the chromosome; Percentage (%)—coverage of the substitution segment.

**Table 3:** List of 36 QTLs identified from DP30-CSSLs.

Traits	QTL	LOD	Position	Spring		Fall		Cloned Gene
				a	ap (%)	a	ap (%)	
TA	*qTA1.1*	*2.4*	C1-26–C1-27	−1.6	9.7	0.7	9.7	
TA	*qTA7.1*	*2.5*	C7-2	−2.4	17.2	−1.2	14	*PROG1* [17]
TA	*qTA9.1*	*2.7*	C9-14–C9-15	−1.4	16.5	−1.4	16.5	*TAC1* [20]
TA	*qTA2.1*	*2.6*	C2-25	0.4	4.3	0.6	7.3	
HD	*qHD11.1*	*3*	C11-4	−5	4.1	−3.72	2.9	
PH	*qPH1.1*	*3.1*	JM1-5	31.8	17.6	30.7	28.8	*Sd1* [37]
PH	*qPH6.1*	*3*	C6-8	28.1	24.6	27.3	25.6	
PH	*qPH7.1*	*2.6*	C7-5	25.9	19.5	26.7	25	
NGPP	*qNGPP3.1*	*2.8*	C3-19	34.2	19.8	33.1	20.1	
NGPP	*qNGPP4.1*	*5.2*	C4-12	25.2	14.6	25	15.2	
NGPP	*qNGPP12.1*	*2.9*	C12-2	40.3	23.3	37.3	22.6	
GWT	*qGWT1.1*	*3.1*	C1-19	−3	10.2	−0.3	11.1	*OsAGPL2* [38]
GWT	*qGWT2.1*	*4.2*	C2-19	−0.2	8	−0.2	7.8	
GWT	*qGWT3.1*	*4.4*	C3-25	−0.3	8.3	−0.3	8.3	
GWT	*qGWT4.1*	*3.4*	C4-15	−0.3	10	−0.3	9.9	
GWT	*qGWT5.1*	*4*	C5-23	−0.3	10	−0.3	10.1	
GL	*qGL3.1*	*3.8*	C3-19	−0.9	9	1	10	
GL	*qGL9.1*	*3.4*	C9-12	−0.9	8.5	−0.9	8.7	*SG1* [39]
GW	*qGW9.1*	*3.2*	C9-8	−0.1	3.2	−0.1	2.7	
GW	*qGW10.1*	*3.1*	C10-3	−0.1	3.4	−0.1	3.1	
GLWR	*qGLWR3.1*	*3.5*	C3-26	0.3	11.4	0.3	11.1	
GLWR	*qGLWR8.1*	*3.9*	C8-2	0.3	10.9	0.3	11.2	
AL	*qAL1.1*	*3*	C1-7	5.6	26.9	5.6	32.7	
AL	*qAL4.1*	*2.7*	C4-8–C4-10	9.8	47.6	10.1	59.3	*An-1* [40]
AL	*qAL4.2*	*4.3*	C4-19	10.3	49.6	10.3	60.8	*An-2* [41]
AL	*qAL8.1*	*2.6*	C8-11	6.8	33.1	6.4	37.7	
AL	*qAL11.1*	*3.9*	C11-2	6.3	30.6	6.9	40.5	
SH	*qSH4.1*	*3.4*	C4-22	−12.5	−8.3	−12	−9	*SH4* [42]
SH	*qSH11.1*	*2*	C11-5~C11-8	−40	−26.7	−42	−27.7	
CT	*qCT1.1*	*2.6*	C1-16	1.3	48.5	1.2	53.3	*OsRAN1* [43]
CT	*qCT2.1*	*2.6*	C2-19	1.2	46.2	0.9	42.9	
CT	*qCT3.1*	*2.9*	C3-2	1.2	45.8	1.1	48.1	
CT	*qCT5.1*	*2.1*	C5-20	1.8	67.3	1.4	65.2	*OsiSAP8* [44]
CT	*qCT6.1*	*2.4*	C6-20	1.8	68.4	1.4	61.4	*OsPYL9* [45]
CT	*qCT10.1*	*3*	C10-3	1.4	51.9	1.4	64.8	
CT	*qCT12.1*	*2.2*	C12-11	1.2	46.8	1	46.5	

List of wild rice QTLs identified in this study. Position—physical position of molecular markers on chromosomes (Mb); length—length of the overlapping part of the lines; a—additive effect; Gene— cloned genes in the overlapping segments. TA— tiller angle; HD—heading date; PH—plant height; NGPP—number of grains per panicle; GWT—1000 grain weight; GL—grain length; GW—grain width; GLWR—grain length to width ratio; AL—awn length; SH—grain shattering; and CT—cold tolerance.

## Supplementary Materials

The following are available online at https://www.mdpi.com/2073-4425/11/9/980/s1, Figure S1: Frequency distributions of quantitative traits in DP30-CSSLs during fall and spring, Figure S2: Phenotypic variation in plant-architecture-related traits and heading date in CSSLs and 93-11, Figure S3: Cold tolerance phenotype of some CSSLs and 93-11 at seedling stages (bar = 10 cm), Figure S4: Phenotypic variation of leaf traits in CSSLs and 93-11, Figure S5: Phenotypic variation of in apiculi, glumes and seed coats in CSSLs and 93-11, Figure S6: The distribution of the 36 QTLs in DP30-CSSLs. The molecular markers are shown on the left and the QTL sites are shown on the right. A QTL overlap indicates that there are two QTL in the same region. Table S1: Whole-genome re-sequencing analysis of wild rice DP30. Table S2: The sequence of DP30-CSSLs molecular markers, Table S3: Substitution segments of DP30-CSSLs population, Table S4: The markers sequences of InDel molecular markers for mapping QTL qCT2.1, Table S5: Genotypes and phenotypes of secondary F_2_ populations.
