# Supplementary material for: Development of Chromosome Segment Substitution Lines (CSSLs) Derived from Guangxi Wild Rice (Oryza rufipogon Griff.) under Rice (Oryza sativa L.) Background and the Identification of QTLs for Plant Architecture, Agronomic Traits and Cold Tolerance
Source: Genes (Basel). 2020 Aug 22;11(9):980. doi: 10.3390/genes11090980 (PMC7564255; doi:10.3390/genes11090980)
Supplement: Supplementary file 1 [file genes-11-00980-s001.zip › Supplementary file 1.docx]

**
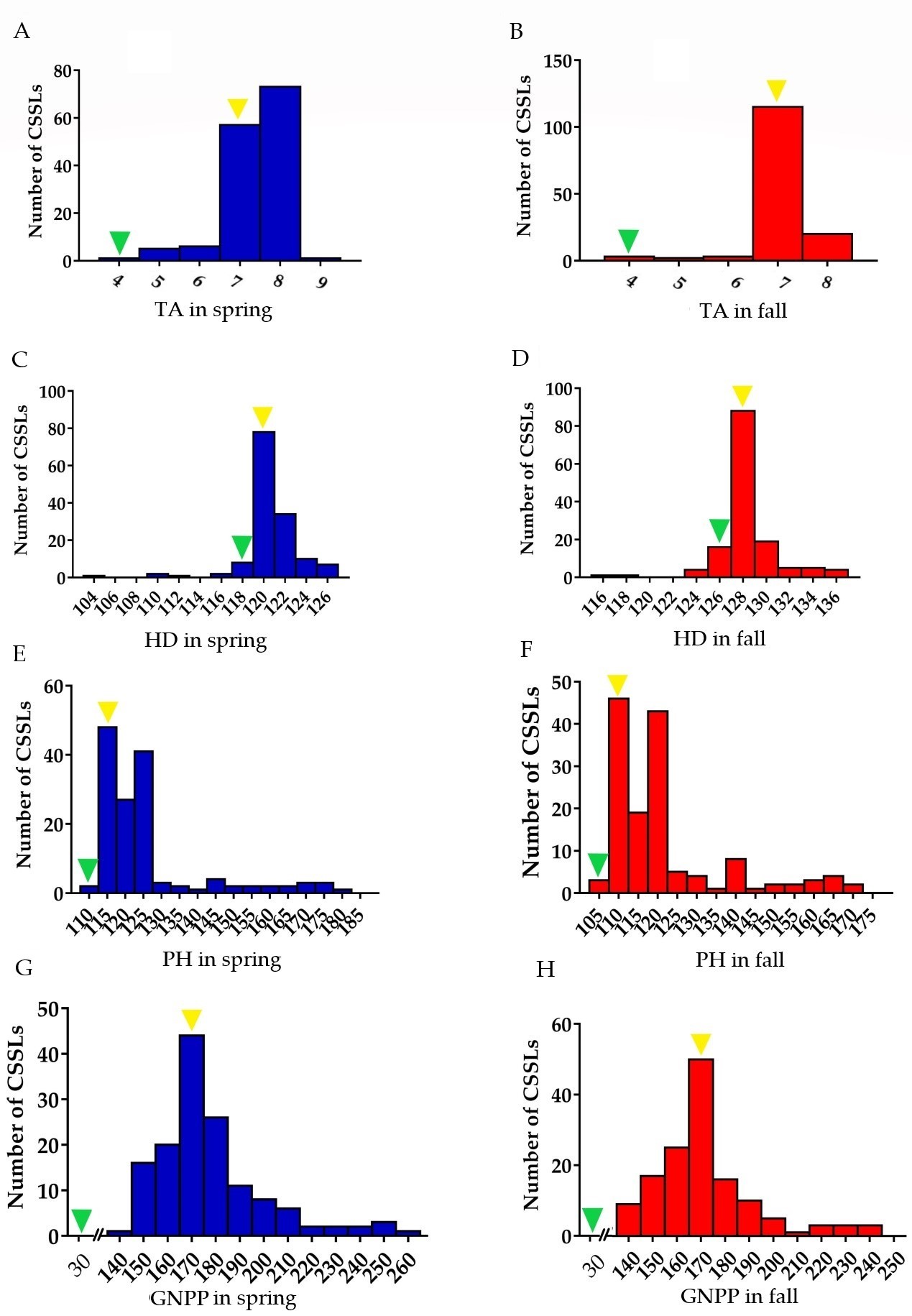

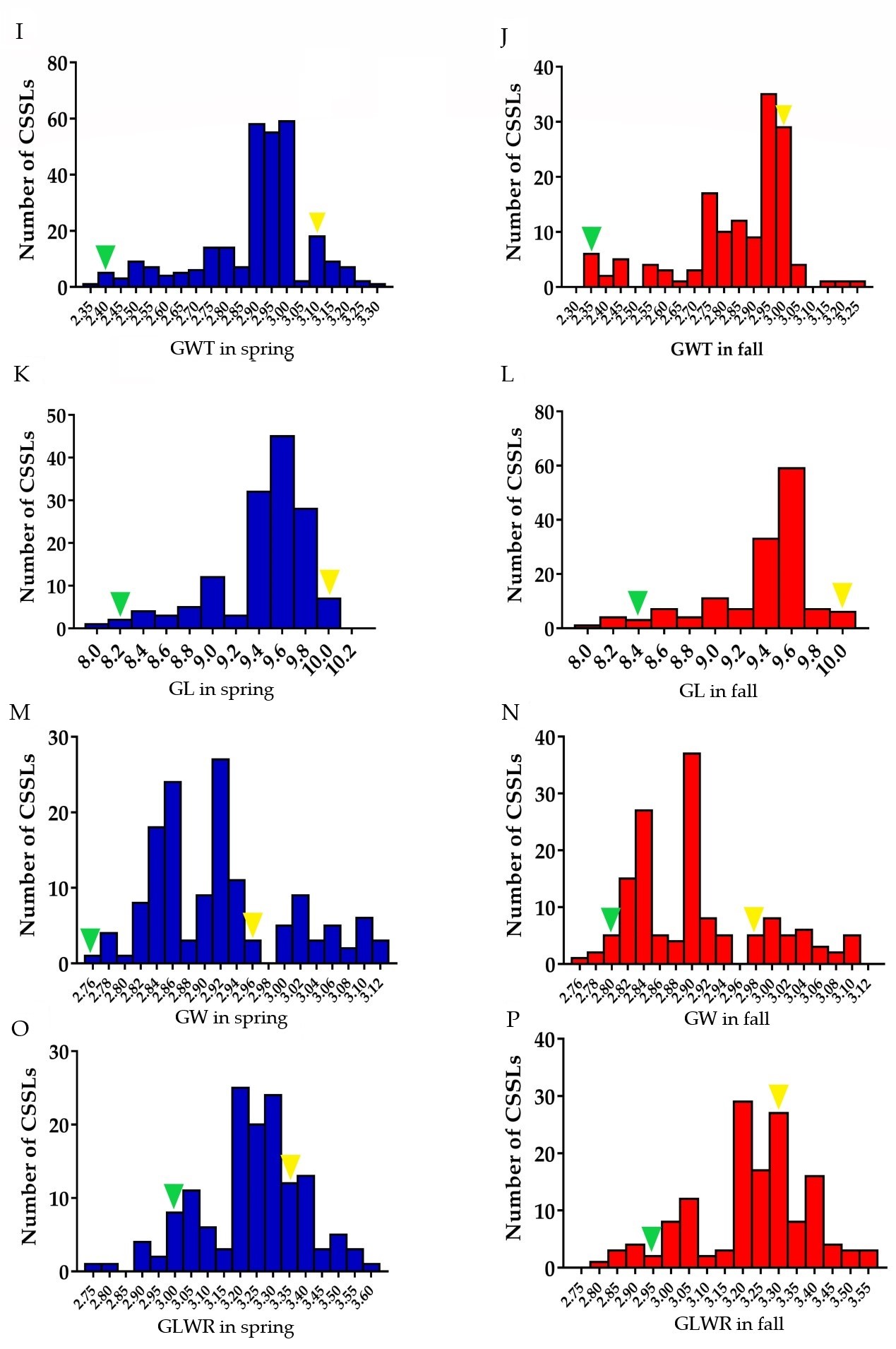
**

**
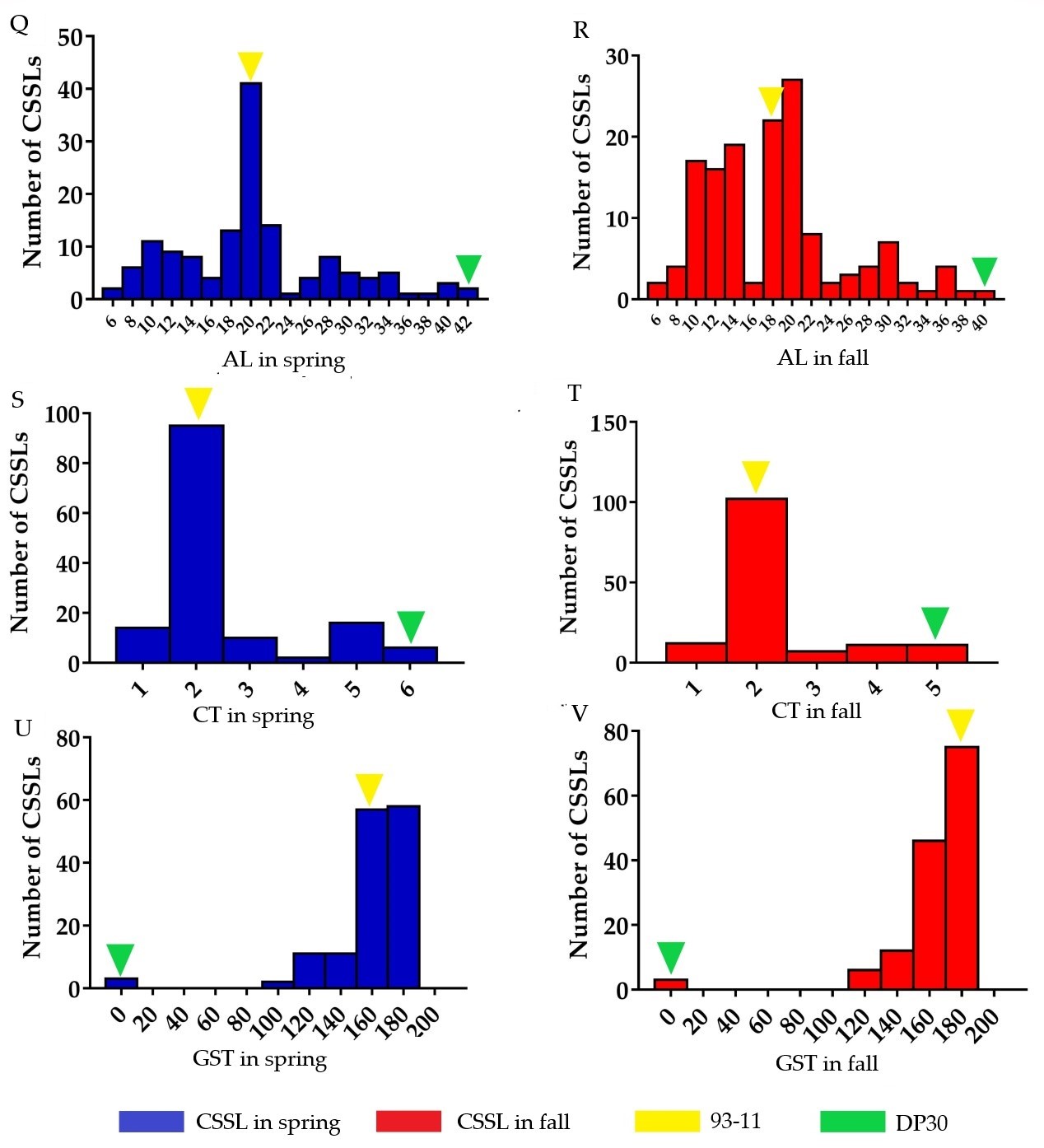
**

**Figure S1.** Frequency distributions of quantitative traits in DP30-CSSLs during fall and spring. **(A, B)** Tiller angle (TA). **(C, D)** Heading date (HD). **(E, F)** Plant height (PH). **(G, H)** Numbers of grains per panicle (NGPP). **(I, J)** 1000 grain weight (GWT). **(K, L)** Grain length (GL). **(M, N)** Grain width (GW). **(O, P)** Grain length to width ratio (GLWR). **(Q, R)** Awn length (AL). **(S, T)** Cold tolerance (CT). **(U, V)** Grain Shattering (GST). Blue box; the value of DP30-CSSLs in spring, Red box; the value of DP30-CSSLs in fall, Yellow arrow: the values of DP30, Green arrow: the values of 93-11.


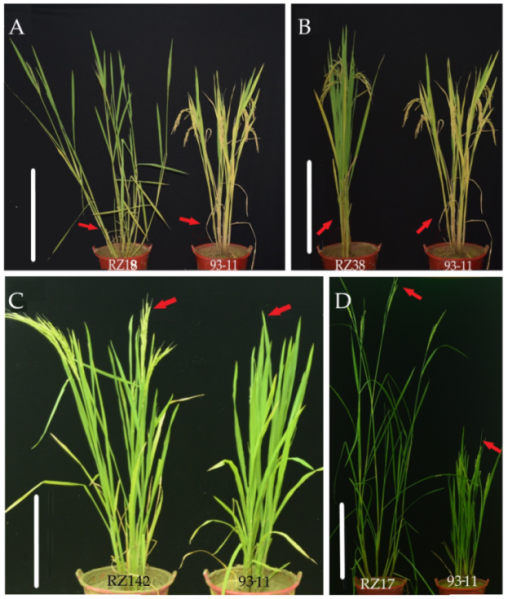


**Figure S2.** Phenotypic variation in plant-architecture-related traits and heading date in CSSLs and 93-11. **(A)** Tiller angle in RZ18 and 93-11, red arrow represents the position of the tiller angle (Bar = 50 cm). **(B)** Tiller angle of RZ38 and 93-11, red arrow represents the position of the tiller angle (Bar = 50 cm). **(C)** Heading date, red arrow represents the position on the rice plane where heading occurred (Bar = 50 cm). **(D)** Plant height, red arrow represents the highest point of rice (Bar = 50 cm).


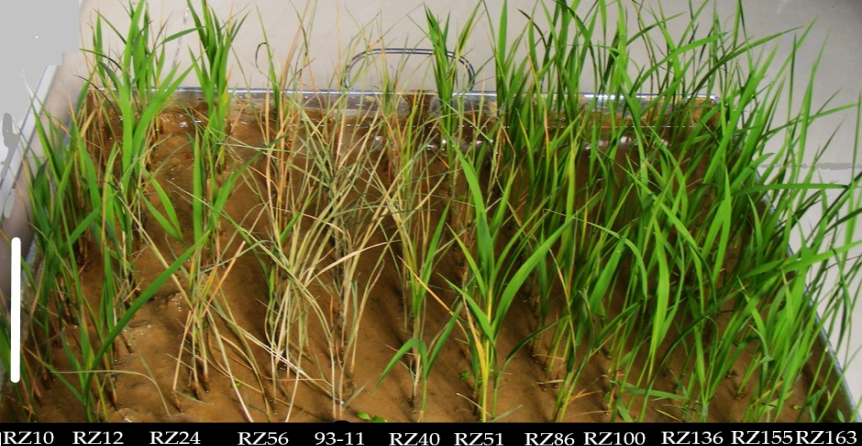


**Figure S3.** Cold tolerance phenotype of some CSSLs and 93-11 at seedling stages (bar=10cm).


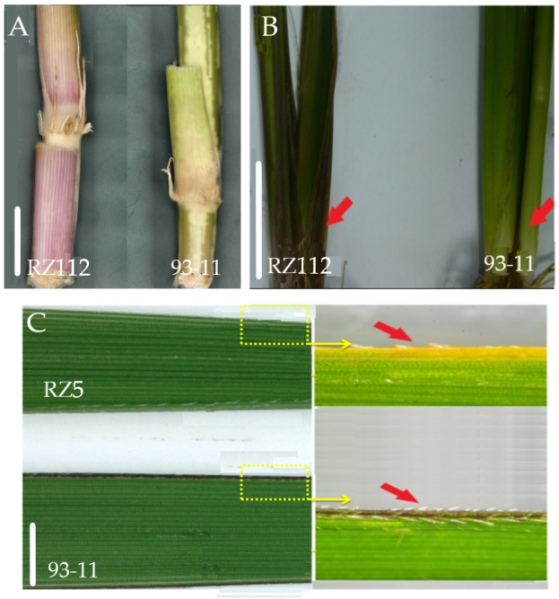


**Figure S4.** Phenotypic variation of leaf traits in CSSLs and 93-11. **(A, B)** Leaf sheath color of RZ133, RZ21 and 93-11 (A: bar = 1cm, B: bar = 10 cm) **(C)** leaf margin color in RZ21 and 93-11 (bar = 2 cm).


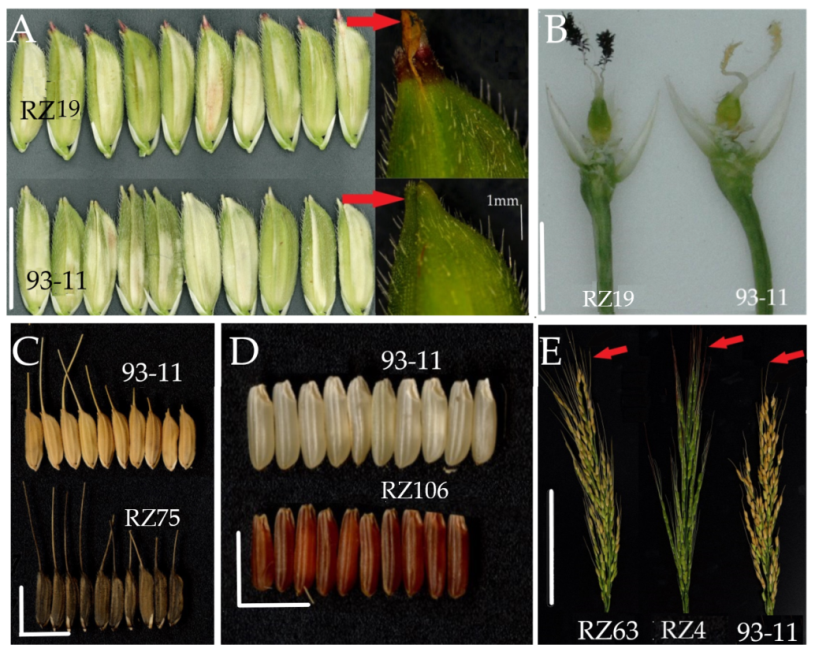

 **Figure S5.** Phenotypic variation of in apiculi, glumes and seed coats in CSSLs and 93-11. **(A)** Apiculus (red arrow) color in RZ18 and 93-11 (bar = 1 cm). (**B)** Stigma color in RZ135 and 93-11 (bar = 1 cm). (**C)** Glume color in RZ87 and 93-11 (bar = 1 cm). (**D)** Seed coat color in RZ105 and 93-11 (bar = 1 cm). (**E)** Awn (red arrows) color in Rz63, RZ130 and 93-11 (bar = 10 cm.


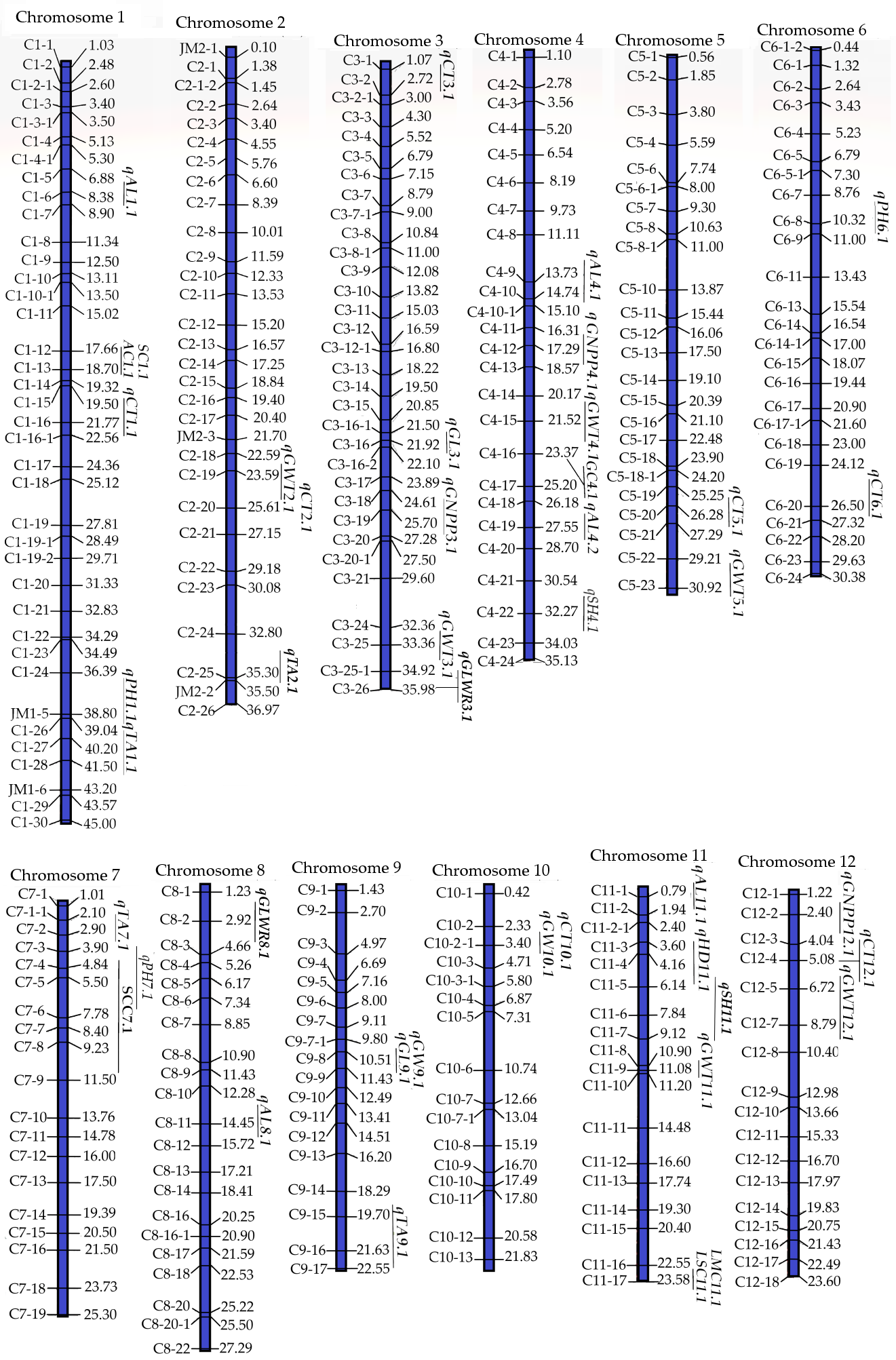


**Figure S6.** The distribution of the 36 QTLs in DP30-CSSLs. The molecular markers are shown on the left and the QTL sites are shown on the right. A QTL overlap indicates that there are two QTL in the same region.
